# A Combined Experimental and Theoretical Study of ESR Hyperfine Coupling Constants for *N,N,N’,N’*-Tetrasubstituted *p*-Phenylenediamine Radical Cations

**DOI:** 10.3390/ijms24043447

**Published:** 2023-02-08

**Authors:** Ronan Gleeson, Cecilie L. Andersen, Peter Rapta, Peter Machata, Jørn B. Christensen, Ole Hammerich, Stephan P. A. Sauer

**Affiliations:** 1 Department of Chemistry, University of Copenhagen, Universitetsparken 5, DK-2100 Copenhagen, Denmark; 2 Institute of Physical Chemistry and Chemical Physics, Faculty of Chemical and Food Technology, Slovak University of Technology, Radlinského 9, 812 37 Bratislava, Slovakia

**Keywords:** ESR, organic radicals, DFT, J-augmented basis sets

## Abstract

A test set of *N,N,N’,N’*-tetrasubstituted *p*-phenylenediamines are experimentally explored using ESR (electron spin resonance) spectroscopy and analysed from a computational standpoint thereafter. This computational study aims to further aid structural characterisation by comparing experimental ESR hyperfine coupling constants (hfccs) with computed values calculated using ESR-optimised “J-style” basis sets (6-31G(d,p)-J, 6-31G(d,p)-J, 6-311++G(d,p)-J, pcJ-1, pcJ-2 and cc-pVTZ-J) and hybrid-DFT functionals (B3LYP, PBE0, TPSSh, ωB97XD) as well as MP2. PBE0/6-31g(d,p)-J with a polarised continuum solvation model (PCM) correlated best with the experiment, giving an *R*${}^{2}$ value of 0.8926. A total of 98% of couplings were deemed satisfactory, with five couplings observed as outlier results, thus degrading correlation values significantly. A higher-level electronic structure method, namely MP2, was sought to improve outlier couplings, but only a minority of couples showed improvement, whilst the remaining majority of couplings were negatively degraded.

## 1. Introduction

Electron spin resonance (ESR)—also commonly known as electron paramagnetic resonance (EPR) spectroscopy—is an important tool in the study and characterisation of organic radical species [1]. Computational investigations aiding such ESR experiments are critical in assessing especially short-lived species that are experimentally difficult to monitor.

**Figure 1:**
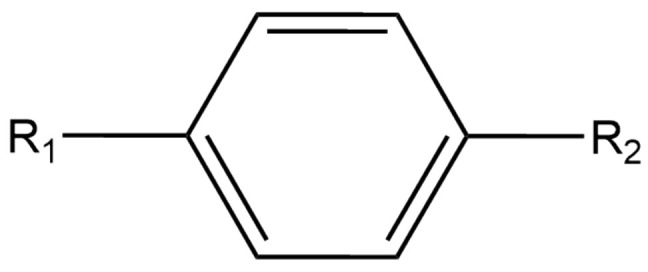
General structure of each *N,N,N’,N’*-tetrasubstituted *p*-phenylenediamine with R${}_{1}$ and R${}_{2}$ defined in Table 1.

A chemically interesting group of molecules that are an exception to this fact are persistent *N,N,N’,N’*-tetrasubstituted *p*-phenylenediamine radical cationic species, the redox properties of which have been explored in detail [2,3]. Applications include materials for solar cells [4,5], artificial photosynthesis [6,7,8], and organic molecular conductors [9], to name a few. The first study of *N,N,N’,N’*-tetrasubstituted *p*-phenylenediamines included ESR electron nuclear double resonance spectroscopy (ENDOR) measurements complemented by density functional theory (DFT) calculations of TMePD, TiPrPD, and BPyrB [10] (For structures and abbreviations, see Figure 1 and Table 1). At the B3LYP/6-31G(d,p) level of theory, hyperfine coupling constants (hfccs) were underestimated by 5–8%, with progress stunted since. Recently, this molecular test set was expanded, and the redox properties [2,11] have been studied in great detail. Substantial progress has been made in the measurement of formal redox potentials with computational analysis verifying experimental potentials with high confidence. Different computational methods have displayed varying degrees of success, with a multi-level G3MP2B3 method producing the highest *R*${}^{2}$ value of 0.9624 but at the cost of a diminished slope and *y*-intercept. DFT/B3LYP with a large 6-311++G(d,p) basis set improved the slope and *y*-intercept parameters significantly, with a marginally depreciated *R*${}^{2}$ value in comparison to G3MP2B3. TPSSh/6-311++G(2d,p) yielded the most robust results with a slightly less than perfect slope. From this, DFT appears to be the obvious choice for this articles’ computational explorations. Additionally, since the initial investigation by Grampp et al. [10], ESR/NMR optimised J-augmented basis sets have become widely available [12,13,14,15,16,17,18,19], hence improvement of correlation with experimental and calculated couplings is promising.

In this present work, we present experimental ESR parameters including 10 g tensor values and 64 hfccs for 11 *N,N,N’,N’*-tetrasubstituted *p*-phenylenediamines obtained by in situ EPR/UV–vis–NIR spectroelectrochemical measurements. In addition, we report hfcc results for a series of DFT calculations aiming to determine a computational protocol suitable for reproducing experimental hfccs (similar to our previous study of *N,N,N’,N’*- tetrasubstituted *p*-phenylenediamine redox potentials [2]). In principle, *N,N,N’,N’*-tetrasubstituted *p*-phenylenediamines are optimal for ESR experimental investigations [20] as ESR-signals are observed only for radical species.

## 2. Results

### 2.1. Experimental Results

All experimentally determined ESR hfccs and g-factors are listed in Table 2 (with DMeAzirA g-factor indeterminable). Experimental ESR results determine a${}_{arom}^{H}$ couplings as a positive value; yet, when compared to ENDOR investigations of similar compounds [21], a negative value is deduced. Therefore, negative experimental a${}_{arom}^{H}$ coupling values will be used in the linear regression studies to correctly align with computed a${}_{arom}^{H}$ couplings. As expected, the g-factor is invariant towards the alkyl substituents, and the value 2.0059±0.0001 is obtained for all compounds. The hfccs for TMePD are a${}^{N}$ = 0.7156 mT, a${}_{arom}^{H}$ = 0.2071 mT, and a${}_{CH_{3}}^{H}$ = 0.6686 mT—differing by +0.0157 mT, +0.0074 mT, and -0.0084 mT, respectively, from reported values in similar literature [10,22,23]. Similar agreements with the literature values are observed for the hfccs of TiPrPD and BPyrB [10]. For each asymmetric compound, two nitrogen atoms are chemically different, as are the hydrogen atoms attached to the central ring, yet it is not possible to experimentally distinguish each individual atom. However, only small changes in each hfcc are observed for these atoms depending on the substituents, and their exact identification is not crucial for this article.

### 2.2. Computational DFT Results

In the following, the main form of statistical analyses for correlating experimental and computed results will be communicated via correlation plots and calculation of suitable quantitative measures such as *R*${}^{2}$ values, slopes (*m*), and *y*-axis intercepts (*c*). The results for the individual molecules, couplings, functionals and basis sets are shown in Tables S1–S11 of the Supplementary Material.

**Figure 2:**
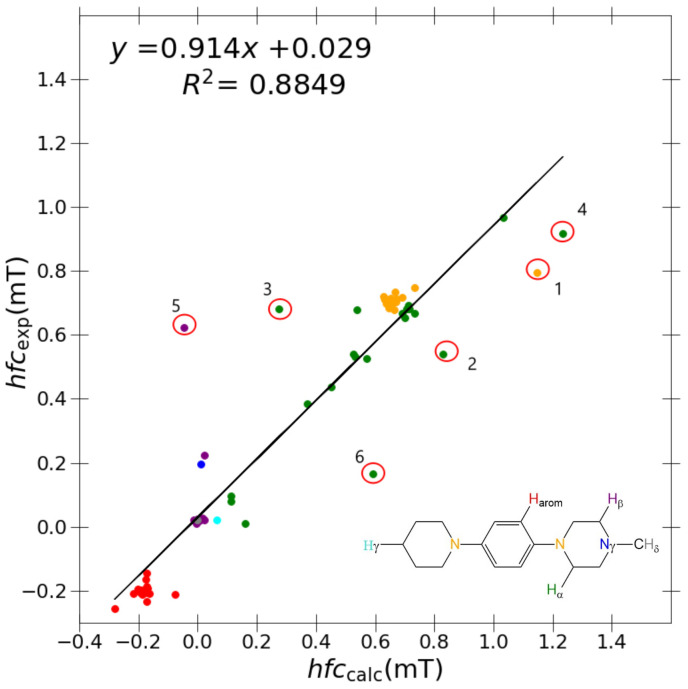
Correlation between experimental and calculated hyperfine coupling constants using B3LYP/6-31G(d,p)/PCM model. Outliers marked with circles are DMeAzirA (1) $a^{N}$, (2) $a_{CH_{3}}^{H}$, and (3) $a_{CH_{2}}^{H}$; DMeAzetA (4) $a_{CH_{2}\alpha }^{H}$ and (5) $a_{CH_{2}\beta }^{H}$; and DMeDiPrPD (6) $a_{CH}^{H}$. The colour coding of the results corresponds to the general *N,N,N’,N’*-tetrasubstituted *p*-phenylenediamine shown in the inset: orange (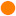): $a^{N}$; blue (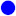): $a_{\gamma }^{N}$; red (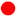): $a_{arom}^{H}$; green (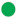): $a_{CH_{2}\alpha }^{H}$; purple (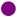): $a_{CH_{2}\beta }^{H}$; cyan (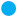): $a_{CH_{2}\gamma }^{H}$; grey (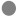): $a_{CH_{3}\delta }^{H}$.

Initially, a study using B3LYP and a relatively small, double-zeta Pople basis set, 6-31G(d,p), was conducted (see Figure 2), yielding *R*${}^{2}$ = 0.8849, slope = 0.914, and *y*-intercept = 0.029 (Table 3) between the calculated and experimental couplings within the collated set of eleven target molecules. Considering that we have included a diverse set of nuclei, the correlation with experiment is relatively satisfactory.

Nevertheless, in Figure 2 we can identify six distinct outlier couplings namely DMeAzirA-(1) $a^{N}$ (2) $a_{CH_{3}}^{H}$ (3) $a_{CH_{2}}^{H}$; DMeAzetA-(4) $a_{CH_{2}\alpha }^{H}$ (5) $a_{CH_{2}\beta }^{H}$ and DMeDiPrPD-(6) $a_{CH}^{H}$ using B3LYP/6-31G(d,p). Here, there exists obvious room for improvement, hence the motivation to trial ESR-optimised “J-style” basis sets. As a preliminary analysis, we tested the “J-style” versions of the popular Pople basis sets, 6-31G(d,p)-J and 6-311++G(d,p)-J [16], DFT-optimised polarization consistent basis sets, pcJ-1 and pcJ-2 [14], and a version of the correlation consistent original “J-style” basis set without extra diffuse functions, cc-pVTZ-J [13]. Shown in Table 3 is the comparison of B3LYP/6-31G(d,p) with B3LYP/“J-style” basis sets comparing each computational protocols regression performance with experimental values to quantitatively assess if enhanced correlation has been achieved.

**Figure 3:**
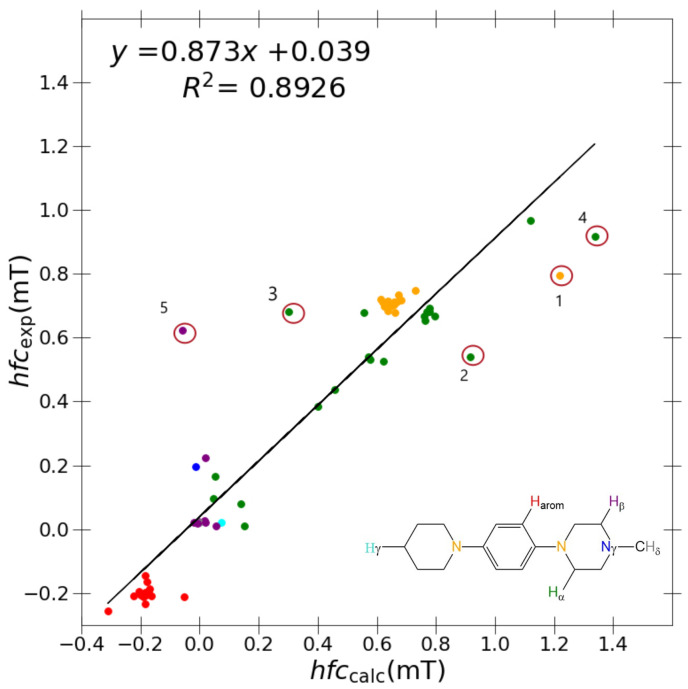
Correlation between experimental hyperfine coupling constants and calculated constants using PBE0/6-31G(d,p)-J/PCM model. Outliers marked with circles are DMeAzirA (1) $a^{N}$, (2) $a_{CH_{3}}^{H}$, and (3) $a_{CH_{2}}^{H}$, and DMeAzetA (4) $a_{CH_{2}\alpha }^{H}$ and (5) $a_{CH_{2}\beta }^{H}$. The colour coding of the results corresponds to the general *N,N,N’,N’*-tetrasubstituted *p*-phenylenediamine shown in the inset: orange (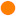): $a^{N}$; blue (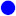): $a_{\gamma }^{N}$; red (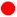): $a_{arom}^{H}$; green (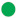): $a_{CH_{2}\alpha }^{H}$; purple (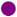): $a_{CH_{2}\beta }^{H}$; cyan (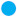): $a_{CH_{2}\gamma }^{H}$; grey (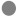): $a_{CH_{3}\delta }^{H}$.

It is quite clear that 6-31G(d,p) still reigns superior over the augmented “J-style” basis sets, retaining a higher *R*${}^{2}$ and slope combined with an impressively lower *y*-intercept value. Comparing the best performing “J-style” 6-31G(d,p)-J basis set with the dominant 6-31G(d,p), one observes the largest dis-improvement in terms of slope and *y*-intercept as we transition to the “J-style” regime. With respect to the *R*${}^{2}$ value, an extra outlier coupling is observed (six couplings in Figure 2 for 6-31G(d,p) compared to five couplings in Figure 3 for 6-31G(d,p)-J), negating some of the positive contributions from other coupling groups to the *R*${}^{2}$ value (and even other correlation parameters).

In terms of the “J-style” basis set performance, 6-31G(d,p)-J fairs best, with a degrading correlation trend observed as basis set size increases towards cc-pVTZ-J. Comparing 6-31G(d,p)-J and cc-pVTZ-J, one observes that all coupling groups become less correlated contributing to a noticeably worse *R*${}^{2}$ value. In contrast, a better slope is observed for cc-pVTZ-J in comparison to 6-31G(d,p)-J, yet such behaviour is attributed to a larger set of under-calculated couplings, namely $a^{N}$ couplings, inadvertently shifting the slope towards unity. Comparing absolute calculated coupling values, 59% of nitrogen couplings become underestimated by 10% or more whilst all other coupling groups remain consistent throughout the “J-style” series, including outlier couplings. Similar trends are followed for basis set series comparisons throughout this article.

Another potential route for improvement follows changing the DFT functional and trialling each functional (TPSSh and PBE0) with a combination of each aforementioned “J-style” basis set. Comparisons between newly tested functionals and “J-style” basis sets with the energetically optimised dominant 6-31G(d,p) basis set are shown in Table 4.

With reference to Table 4, the best performing basis set and functional combination is PBE0/6-31G(d,p)-J/PCM with an *R*${}^{2}$ = 0.8926, *m* = 0.873 and *c* = 0.039. Arguably, a higher *R*${}^{2}$ value of 0.8957 (vs. 0.8926) is recorded for TPSSh/6-31G(d,p)-J/PCM, but the strength of correlation (i.e., slope) diminished (*m* = 0.845 vs. 0.873) considerably in comparison to PBE0/6-31G(d,p)-J/PCM. The relative error (i.e., intercept) for each model is equal at *c* = 0.039. Therefore, PBE0/6-31G(d,p)-J/PCM is deemed a superior model due to a noticeably better strength of correlation. The relative lack of description within the 6-31G(d,p)-J basis leads one to conclude that error cancellation effects may be of total dominance in this study. The inherent issue may arise from the error of the approximated E${}_{XC}\left[\rho \right]$ functional cancelling with the large, direct basis set error of the smallest 6-31G(d,p)-J basis set.

## 3. Discussion

Literary articles [10,24] disseminating the calculation of hfccs typically employ small basis sets in hybrid-DFT–ESR calculations, concluding that an error cancellation approach may be counter-intuitively optimal in this instance. With reference to TPSSh and B3LYP, the gap between the worst and best described basis sets, 6-31G(d,p)-J and cc-pVTZ-J, respectively, is more evident, with PBE0 actually maintaining a more negligible gap between 6-31G(d,p)-J and cc-pVTZ-J. In retrospect, the PBE0 functional best abides with a logical a priori prediction of the largest cc-pVTZ-J basis set best aligning calculated results with experimental coupling constants. From this, one may conclude that the PBE0 functional may suffer least from functional error by retaining the most satisfactory cc-pVTZ-J results.

A marginal improvement of ≈1% is seen in Figure 3 using PBE0/6-31G(d,p)-J/PCM model for calculating a${}_{{CH_{2}}_{\alpha }}^{H}$ and a${}_{{CH_{2}}_{\beta }}^{H}$ couplings in comparison to B3LYP/6-31G(d,p), hence promoting a greater *R*${}^{2}$ value. However, outlier couplings degraded further, giving a marginally poorer correlation slope and *y*-intercept as shown in Table 4. It is difficult to conclude which results are better between each model, but based on the hierarchical importance of the *R*${}^{2}$ value, the PBE0/6-31G(d,p)-J/PCM model is subjectively better.

As seen in Table 3, two opposing trends also appear here in Table 4 as we correlate the change in basis set size with the linearly regressed parameters; *R*${}^{2}$, slope and *y*-intercept across the three functionals. Trending the change in *R*${}^{2}$ and *y*-intercept, one observes improved values for each parameter as basis set size shrinks. Conversely, when examining the change in slope, a value closer to unity is calculated as the basis set size increases. The fact that underestimation of, namely $a^{n}$ couplings, produces seemingly opposing trends eludes a causal explanation of these trends.

As shown, the results are not satisfactorily intuitive—and saying 6-31G(d,p) is a “better” basis set than cc-pVTZ-J is incorrect. One potential source of error within this works’ computational model lies with the implementation of an implicit solvation model, specifically the polarised continuum model (PCM). It is hard to judge how much this may effect the accuracy of the calculation but for some systems this may be considerable. Instead, one could implement an explicit solvation model to better replicate an experimental solvation environment. As shown in Table 5, the improvement is considerable transitioning from a system absent of solvation to a system with implicit solvation, hence this more accurate model may bring potential improvements. In reality, due to the high computational cost associated with an explicit solvation model paired with this studies relatively large molecules, an explicit route may not be advised.

### 3.1. Basis Set Sensitivity and Robustness

It is also of interest to examine correlation parameter’s sensitivity and robustness to increasing basis set size for each functional. Firstly, regarding B3LYP, *R*${}^{2}$ and slope change by less than ±2.1%, whilst PBE0 is best, at less than ±1.5%. Meanwhile, TPSSh functional robustness is poorest with changes of less than ±3.1%, concluding that TPSSh sensitivity to change in basis set size is greatest. All functionals show quite poor robustness in terms of the *y*-intercept parameter; however, these values are truly satisfactory as they near zero, hence arguments of robustness may be deemed redundant.

**Figure 4:**
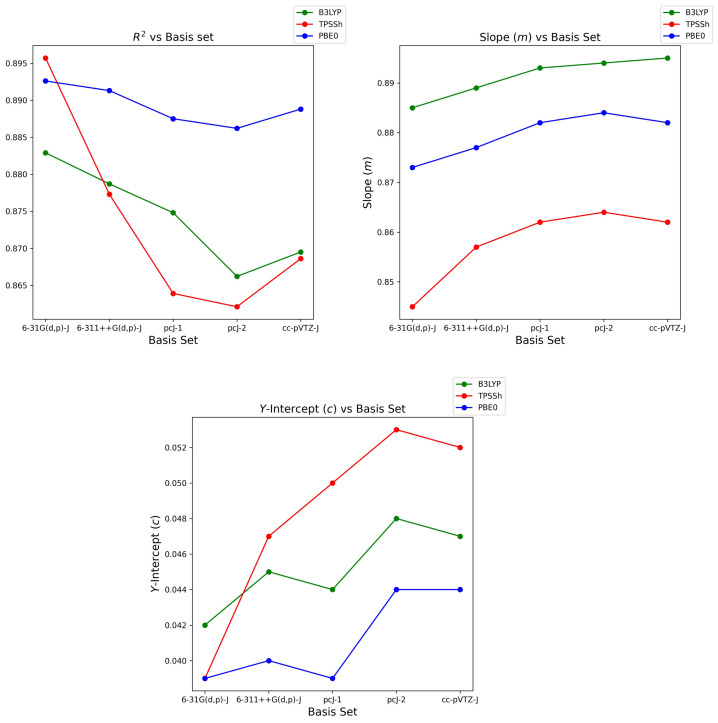
Trend in *R*${}^{2}$, slope (*m*) and *y*-intercept (*c*) parameters transitioning along each basis set for B3LYP, TPSSh and PBE0 functionals.

Examining cc-pVTZ-J for all functionals, the trend in *R*${}^{2}$ and *y*-intercept (except PBE0) improve when transitioning from pcJ-2, suggesting that couplings begin to recover and correlate marginally better as shown in Figure 4. Shown also is the depreciating trend in slope (except B3LYP) as we transition from pcJ-2 to cc-pVTZ-J. From this, one concludes that overall coupling correlation recovers, yet calculated couplings remain overestimated when the largest cc-pVTZ-J basis set size is realised.

### 3.2. Examining Outliers

Common to all “J-style” basis set models tested is the constant set of detrimental outliers degrading calculated coupling quality significantly; hence, an analytic effort is applied hereafter for diagnosing the origin of such erroneous behaviour.

**Figure 5:**
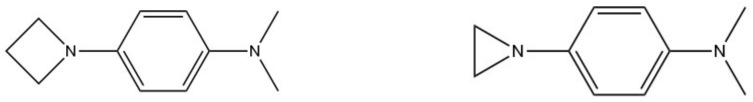
DMeAzetA (**left**) and DMeAzirA (**right**) molecular structures.

As shown in Figure 3, problematic outliers remain, drastically depreciating the quality of the *R*${}^{2}$ value (0.873), slope (0.8926), and *y*-intercept (0.039) within the best-performing “J-style” system PBE0/6-31G(d,p)-J. Regardless of the basis set/functional deployed, these outliers still stem from two molecules exclusively for all “J-style” basis set calculations: DMeAzirA and DMeAzetA (shown in Figure 5). From initial observations, this is an issue pertaining to strained fragments within each molecular structure that are not treated correctly by any hybrid-DFT functional and/or basis set tested.

From a structural standpoint, each molecule shares a highly strained N-ring that projects significant problems onto the coupling calculation for each group present within the ring—with couplings being either drastically over/under-estimated. Four out of five outliers are present within each strained fragment, with the fifth outlier $a_{CH_{3}}^{H}$ attached to the non-cyclic-nitrogen in DMeAzirA. On average, couplings appeared less correlated with experimental values in DMeAzirA compared to DMeAzetA, concluding that increased straining effects have a global impact on couplings within the molecule.

In comparison, couplings within the slightly less strained DMeAzetA system are better correlated with experimental results, yet still exhibit major outlier behaviour within the collated set of calculated couplings. Other couplings that are deemed “non-outliers” in the collated set reflect that they are independent of minor perturbations of other substituents in the instances that these perturbations do not originate from a strained ring fragment.

No other N-strained ring is present in this subset of *N,N,N’,N’*-tetrasubstituted *p*-phenylenediamines, concluding that this is an issue of ineptness within the computational model’s ability to treat couplings associated with highly strained N-ring systems. From molecular structure theory, the interior angle between atoms in three/four member rings is much less than ideal (compared to similar five or six member rings), forcing an increased overlap of electron density. In strained systems, some “wiggle” room is allowed (maybe forcing a group out of the plane) to alleviate this overlap, so a different conformation may be optimal for the DFT functional and/or basis set used.

Referencing Table 6, a clear depreciating trend in correlation parameters is shown as one transitions from three-member towards six-member ring systems when assessing individual molecular performance with PBE0/6-31G(d,p)-J. DMeAzirA exhibits the largest ring strain within the three-member ring, imposing a poor coupling correlation, whereas the four-member DMeAzetA exhibits considerably better results, albeit still performing poorly within the set of the eleven molecules tested. In contrast, BPipB showcases the best correlation, with a marginal improvement observed transitioning from a five-member ring to a six-member ring. Such results reiterates the detrimental impact that ring strain imposes on smaller (three–four) ring systems.

Of interest also was examining the change in correlation values upon removal of both problematically strained systems, shown in Table 7. As expected, all regression parameters improved significantly for all functional and basis set combinations, with similar trends to those observed in Table 4. Most evident is the superior correlation trend attained by the smallest basis set-6-31G(d,p)-J, largely due to suspected basis set and functional error cancellation. As the basis set size increases towards cc-pVTZ-J, experimental and calculated values deviate due to calculated couplings becoming under-calculated, generally, with PBE0 functional retaining greatest robustness towards basis set size increase.

These strained fragments were also deemed problematic in the calculation of oxidation potentials in a related article [2], yet it was observed that a higher-level electronic structure model treated such outliers appropriately, thus improving results significantly. In this article, a similar approach is trialled to improve DFT results by using a slightly more sophisticated model such as MP2. Due to the inordinate scaling of CCSD or CCSD(T), MP2 with N${}^{5}$ (where N is one electron basis function) scaling possesses a satisfactory middle ground between computation time and accuracy.

### 3.3. Computational MP2 Analysis

**Figure 6:**
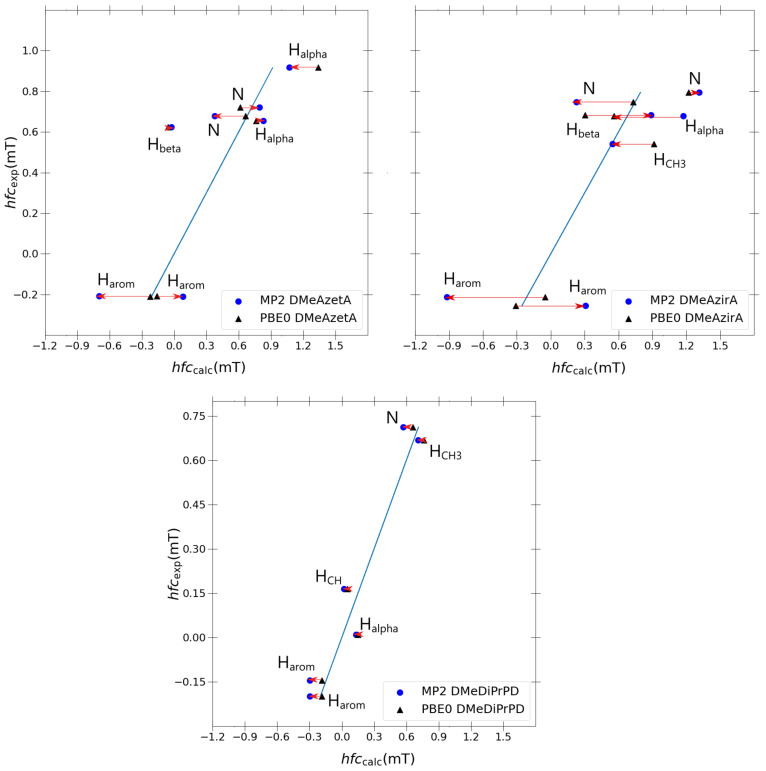
Individual PBE0 vs. MP2 hyperfine coupling constant comparisons for outlier molecules DMeAzetA, DMeAzira and satisfactory molecule DMeDiPrPD using the 6-31G(d,p)-J basis set. Fitted blue line in each graph is the ideal fit line for calculated results given as *y* = *x*.

Taking inspiration from the success of the double-zeta Pople basis sets in the previous section, it was decided to re-optimise each structure using MP2/6-31G(d,p), utilising a J-augmented 6-31G(d,p)-J basis set for each MP2 single-point hfcc calculation. Three molecules were chosen for sampling the quality of MP2, namely the two outlier molecules (DMeAzetA and DMeAzirA) and one molecule that exhibited an excellent correlation with experimental results (DMeDiPrPD). A 1:1 comparison of each coupling with the blue ideal fitting line (*y* = *x*) is shown in Figure 6 to observe the improvement, if any, that is gained transitioning from PBE0 to MP2.

Comparing changes transitioning from PBE0 to MP2 for each molecule in Figure 6, it is apparent that the discrepancies between experimental and calculated values increase significantly as we compare the well-behaved DMeDiPrPD system with the ill-behaved DMeAzetA and DMeAzirA systems. This behaviour is quite possibly a direct effect of the strained rings—most evidently seen in DMeAzirA and reducing in effect as we transition to DMeAzetA—with DMeDiPrPD behaving appropriately due to an absence of such straining effects. Such large changes suggest that strained compounds are more sensitive to how electron correlation is treated compared to non-strained compounds, as one might have expected. However, the large changes attained from going to MP2 do not improve on the quality of correlation as was the case in the previous study of redox potentials related to such *N,N,N’,N’*-tetrasubstituted *p*-phenylenediamines [2].

Regarding individual coupling group comparisons, MP2 always gives poorer $a_{aromatic}^{H}$ couplings in comparison to PBE0, with a similar trend observed for a${}^{N}$ couplings (except for one a${}^{N}$ coupling in DMeAzetA). On average, $a_{CH_{2}\alpha }^{H}$ couplings are split 50:50 between each model, with $a_{CH_{2}\beta }^{H}$ and $a_{CH_{3}}^{H}$ couplings actually improving from PBE0 to MP2. One $a_{CH}^{H}$ coupling was present in favour of PBE0. From this, one can conclude that particular coupling groups are degraded when transitioning from PBE0 to MP2, but others may or may not improve.

The question arises now whether the changes going from PBE0 to MP2 calculations are due to changes in geometry upon performing an MP2 geometry optimization or else due to MP2 calculation of spin density at the nuclei. Hence, it is of interest to compare the hfcc results of a single-point PBE0/6-31G(d,p)-J and MP2/6-31G(d,p)-J calculation with both retaining MP2/6-31G(d,p) optimised geometries for each outlier molecule, DMeAzetA and DMeAzirA.

**Figure 7:**
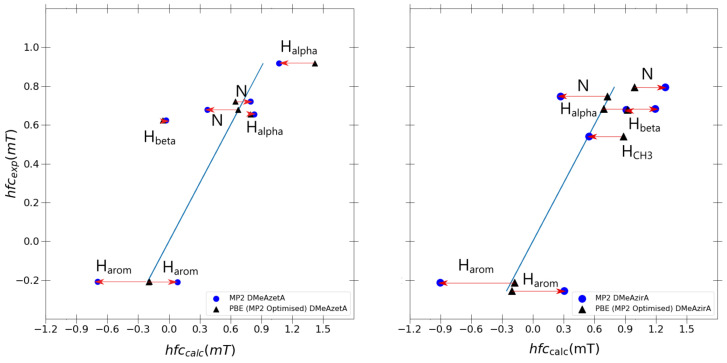
Individual PBE0 vs. MP2 hyperfine coupling constant comparisons for outlier molecules DMeAzetA and DMeAzira with MP2/6-31G(d,p) optimised geometries. Fitted blue line in each graph is the ideal fit line for calculated results given as *y* = *x*.

As shown in Figure 7, one observes that the PBE0 hyperfine couplings remain stagnant compared to Figure 6 differences between PBE0 and MP2 calculated values originating solely from the differences in the spin-density in the hfcc MP2 single-point calculation. Strained effects are still in total effect in both MP2 and PBE0 optimised systems for DMeAzetA and DMeAzirA, with the geometry change between PBE0 and MP2 contributing little to no effect towards improved hfccs (when comparing each molecules performance in Figure 6 and Figure 7). Therefore, the following regression analysis will be based with Figure 6 in mind.

The usual linear regression model is used in Table 8 to compare *R*${}^{2}$, slope and *y*-intercept values retained by MP2 and PBE0. For ease of comparison, both problematic systems and a model DMeDiPrPD system (that achieved satisfactory results) are used to statistically determine if any improvement is gained transitioning from the best performing DFT functional PBE0 to a higher-level method such as MP2.

Evidently, PBE0 remains superior to MP2 for DMeAzetA and DMeDiPrPD as the *R*${}^{2}$, slope, and *y*-intercept retain better values. With respect to DMeAzirA, one actually sees an improved *R*${}^{2}$ value, whereas a quite drastic degradation in slope and *y*-intercept is concurrently observed when transitioning from PBE0 to MP2. These opposing correlation trends for DMeAzirA are explained simply by observing the holistic data point distribution with respect to the fitted line in each separate plot of MP2 and PBE0 in Figure 6. A closer to linear relationship is seen with a superior *R*${}^{2}$ for MP2. Yet, factoring a depreciated slope and *y*-intercept, one concludes that there is a large error from the true fitted line. Moreover, this error originates from the overestimation of calculated couplings at the MP2 level of theory, which is clearly seen upon comparison with corresponding absolute experimental values.

A reference calculation was also performed (Table 8) using ωB97XD optimised using MP2/6-31G(d,p) and a single-point calculation ran with a 6-31G(d,p)-J basis set and PCM solvation model. Surprisingly, DMeAzirA improved significantly over MP2 and PBE0 showing signs for improved correlation upon realisation of the ωB97XD functional. Meanwhile, DMeAzetA and DMeDiPrPD correlation values remain stationary in comparison to MP2 and PBE0. In order to compare this result when functionals previously tested, it was necessary to perform a similar single-point calculation with an ωB97XD/6-31G(d,p) optimised geometry for each of the three test molecules. It is immediately apparent that correlation parameter performance degrades significantly for DMeAzirA in comparison to the MP2/6-31G(d,p) optimised equivalent calculation. Thus, this result falls in line with similar calculations using B3LYP, TPSSh and PBE0 functionals.

As a means to further consolidate discussion of the MP2 results, it was of interest to run a simple single-point calculation using the CFOUR [25] quantum chemistry program. An MP2 calculation was run with an MP2/6-31G(d,p) optimised geometry without solvation parameters and compared to an equivalent calculation run in the Gaussian program. CFOUR by implementation only prints the spin density of each nuclei in the output, hence an extra conversion is needed to calculate the isotropic hfcc a${}_{iso}$ of each nuclei N:

(1) $$a_{iso}\left(N\right)=\frac{4}{3}\pi {S_{z}}^{-1}g_{e}g_{N}\beta_{e}\beta_{N}\rho \left(\vec{R_{N}}\right)$$

where $S_{z}$ is the total spin expectation value, g${}_{e}$ and g${}_{n}$ are the electron and nuclear g-factors, and $\beta_{e}$ and $\beta_{N}$ are the electron and nuclear magnetrons. $\rho (\vec{R_{N}})$ is the nuclear spin density. As shown in Table 5, the results are significantly similar to conclude the MP2 results presented in Table 8 are consistent. Shown also are the DMeAzetA experimental results, which are in much better agreement with MP2 Gaussian results in a solvated environment, further exemplifying the critical improving effect a solvation model provides.

Therefore, to calculate *N,N,N’,N’*-tetrasubstituted *p*-phenylenediamines hfccs, using MP2 would be irrational as the benefit is only marginal for a subset of couplings taking into consideration also the extra cost with respect to hybrid DFT methods. The final synopsis would be to therefore continue with PBE0/6-31G(d,p)-J/PCM for calculating hfccs of *N,N,N’,N’*-tetrasubstituted *p*-phenylenediamines, but a case can be made for using ωB97XD with an MP2-optimised geometry, especially for improving couplings in the strained DMeAzirA molecule.

## 4. Materials and Methods

In the experiment outlined, ESR and UV–vis–NIR measurements are recorded simultaneously, hence both will be referenced within the experimental procedure. Disseminated results from this experiment concentrate solely on ESR measurements and computed results; hence, UV–vis–NIR results are not discussed. Detailed syntheses of each compound have been previously described in detail [2]. Furthermore, compounds were kept under argon during travel to Bratislava, Slovakia and until used for the ESR experiments. The purity of the compounds was then checked by cyclic voltammetry in Bratislava, Slovakia.

### 4.1. Experimental Procedure

Samples for in situ ESR/UV–vis–NIR spectroelectrochemical experiments were prepared by dissolving a weighted amount of electroactive solute and supporting electrolyte (Bu${}_{4}$NPF${}_{6}$) in argon degassed acetonitrile. The concentrations of the solute and the electrolyte were 1 mM and 0.2 M, respectively. All cyclic voltammograms were recorded under argon atmosphere in the potential range of the solute, and the potentiostat triggered the ESR and UV–vis–NIR spectrometers in order to synchronize measurements. The spectroelectrochemical cell (0.1 mm path length) was specially designed for the experiment [26] and suitable for an optical transmission ESR resonator (ER 4104 OR-C 9609) of an EMX ESR spectrometer. The working electrode was a laminated Pt mesh with a small hole in the foil coincident with the light beam, which limited the active surface area of the electrode. A Pt wire auxiliary (counter) electrode and a Ag wire pseudoreference electrode were used. The optical ESR resonator cavity was connected to the diode-array UV–vis–NIR spectrometer Avantes Avaspec (Avantes, Apeldoorn, The Netherlands) by optical fibres. A deuterium–halogen lamp DH 2000 (Sentronic, Schleswig-Holstein, Germany) was used as a light source. UV–vis–NIR spectra were processed by the proprietary AvaSoft 7.7 software package, and the experimental ESR spectra were analysed and simulated using the Bruker softwareWinEPR and SimFonia, respectively. The g-values of the radical cations were determined by using MgO (Mn${}^{2+}$) as an internal standard.

### 4.2. Computational Procedure

Standard one-electron energetically optimised Gaussian basis sets are, in general, not well suited for calculation of hfccs. Conceptually, ESR-optimised basis sets aim to better quantify the true electron density near the nucleus, better approximating the isotropic Fermi-contact term as a result. The Fermi-contact mechanism acts in a coupling regime between electronic spin and the magnetic dipole of the nucleus—with the observed splitting distance between peaks in spectra defined as the magnitude of the hfcc. An anisotropic spin-dipolar interaction also constitutes part of this interaction but is averaged to zero in a solvated environment.

The primary computational goal included modifying the electronic structure calculation to improve hfccs by trialling five ESR-optimised J-augmented basis sets; 6-31G(d,p)-J [16,27,28], 6-311++G(d,p)-J [16,29], pcJ-1 [14], pcJ-2 [14] and cc-pVTZ-J [12,13,30]. As a secondary objective, three hybrid DFT functionals (B3LYP [31,32,33], TPSSh [34,35], and PBE0 [36,37]) were tested in correspondence with the aforementioned ESR-optimised basis sets, and their accuracies were individually assessed via a least-squares linear regression approach. A reference calculation was also run using ωB97XD [38] to compare accuracy with PBE0 and MP2 for strained and non-strained molecules. B3LYP, TPSSH, and ωB97XD functionals have been selected based on their successes in a similar study reproducing oxidation potentials for this family of compounds [2]. Furthermore, the PBE0 functional was selected based on its success predicting hfccs in studies of organic radical species [39] and d-block metals [40,41]. Møller–Plesset second-order perturbation theory (MP2) calculations using these five basis sets were also tested.

Achieving a higher resolution of the electronic density around the nucleus through the use of very-high-exponent s-functions within each basis set is quite straight forward. Yet, true replication of experimental couplings still eludes researchers as other factors such as electron correlation, basis set size, or even relativistic effects always complicate matters. Furthermore, computing discrete solvation effects [42] is neither cheap or trivial, with vibrational averaging [43] also non-negligible in some cases. The latter was not analysed as a method for how to replicate solvation effects in a vibrationally averaged regime has yet to be disseminated. Instead, a polarised continuum solvation model (PCM) provided a satisfactory middle point. Following this, the mantra of this study was not to replicate experimental results with total accuracy, but to provide a clear rationalization of hfcc trends and link outlier results to possible structural anomalies.

Computationally, each target structure underwent a full geometrical optimisation with the same computational model as the calculation of the hfccs with the exception of using standard energetically optimised basis sets instead, i.e., the “non-J augmented” forms. Afterwards, hfccs were calculated within a single-point calculation using the “J-augmented” version of each basis set. In every calculation, the polarised continuum (PCM) solvation model (Gaussian09 keyword; IEFPCM) was deployed replicating fundamental solvation in an approximated framework. All calculations were carried out with the GAUSSIAN09 [44] program. All hfccs are given in milliteslas, mT (1 mT = 10 Gauss).

## 5. Conclusions

In essence, we have presented experimental values of the ESR hyperfine coupling constants of 11 structurally related *N,N,N’,N’*-tetrasubstituted *p*-phenylenediamines. As a reference, we have tested the performance of different computational protocols using a large set of 64 hyperfine couplings consisting of 17 nitrogen and 47 hydrogen nuclei. We concentrated mostly on DFT utilising three exchange correlation functionals—B3LYP, PBE0, and TPSSh—and also presented MP2 calculations for selected systems. Furthermore, we have investigated the performance of “J-style” basis sets which are specially optimised for the calculation of coupling constants.

Overall, calculated values correlated well with experimental values in the scenario that outlier DMeAzetA and DMeAzirA hfccs were excluded from the regression analysis. The usage of “J-style” ESR-optimised basis sets also retained varying degrees of success, with the general trend of increasing basis set size counter-intuitively invoking a degradation of hfcc quality as we moved from a small 6-31G(d,p)-J basis set towards the largest cc-pVTZ-J set. Amongst the set of hybrid DFT functionals tested, PBE0 performed marginally best, hence uniting PBE0 and 6-31G(d,p)-J as the best performing hybrid DFT functional and basis set, respectively, within a PCM solvated environment. Moreover, a transition to MP2 as the electronic structure method yielded a large change in results, especially for both strained systems DMeAzetA and DMeAzirA, yet in a direction that provided no discernible improvement. Hence, PBE0/6-31G(d,p)-J is the choice of electronic structure method and basis set, respectively, within a PCM solvated environment for this set of 11 *N,N,N’,N’*-tetrasubstituted *p*-phenylenediamines. 

## Figures and Tables

**Table 1:** All side R-Groups of each *N,N,N’,N’*-tetrasubstituted *p*-phenylenediamine studied.

Acronym	R${}_{1}$	R${}_{2}$	Acronym	R${}_{1}$	R${}_{2}$
TMePD	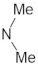	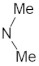	DMeAzirA	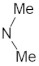	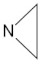
DMeDEtPD	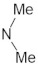	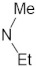	DMeAzetA	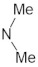	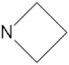
DMeDiPrPD	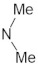	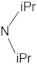	DMeMorphA	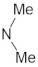	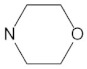
TiPrPD	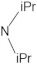	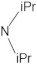	DMePiprzA	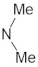	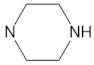
BPyrB	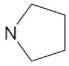	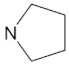	BPipB	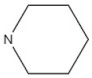	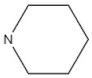
		DMeMePiprzA	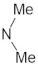	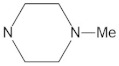	

**Table 2:** Experimental hyperfine coupling constants (mT) determined from ESR spectra.

Compound		Exp	Compound		Exp	Compound		Exp
TMePD	a${}^{N}$	0.7156	BMorphB	a${}^{N}$	0.6988	DMeDiPrPD ${}^{a}$	a${}^{N}$	0.7119
g-factor = 2.0059	a${}_{arom}^{H}$	(-) ${}^{c}$ 0.2071	g-factor = 2.0059	a${}_{arom}^{H}$	(-) ${}^{c}$ 0.2072	g-factor = 2.0059	a${}_{arom}^{H(1)}$	(-) ${}^{c}$ 0.1979
	a${}_{CH_{3}}^{H}$	0.6686		a${}_{CH_{2}\alpha }^{H}$	0.5397		a${}_{arom}^{H(2)}$	(-) ${}^{c}$ 0.1446
				a${}_{CH_{2}\beta }^{H}$	0.2240		a${}_{CH_{3}}^{H}$(Me)	0.6678
							a${}_{CH_{3}}^{H}$(iPr)	0.0091
DMeDEtPD ${}^{a}$	a${}^{N}$(I)	0.6983	DMeAzirA ${}^{a}$	a${}^{N}$(I)	0.7472		a${}_{CH}^{H}$	0.1646
g-factor = 2.0059	a${}_{arom}^{H}$(I)	(-) ${}^{c}$ 0.1648	g-factor ${}^{b}$	a${}_{arom}^{H}$(I)	(-) ${}^{c}$ 0.2559			
	a${}_{CH_{3}}^{H}$(I)	0.6814		a${}_{CH_{3}}^{H}$(I)	0.5402	DMeAzetA ${}^{a}$	a${}^{N}$(I)	0.7200
	a${}^{N}$(II)	0.7030		a${}^{N}$(II)	0.7947	g-factor = 2.0059	a${}_{arom}^{H}$(I)	(-) ${}^{c}$ 0.2087
	a${}_{arom}^{H}$(II)	(-) ${}^{c}$ 0.1959		a${}_{arom}^{H}$(II)	(-) ${}^{c}$ 0.2122		a${}_{CH_{3}}^{H}$(I)	0.6541
	a${}_{CH_{2}}^{H}$(II)	0.3835		a${}_{CH_{2}}^{H(1)}$(II)	0.6783		a${}^{N}$(II)	0.6791
	a${}_{CH_{3}}^{H}$(II)	0.0102		a${}_{CH_{2}}^{H(2)}$(II)	0.6826		a${}_{arom}^{H}$(II)	(-) ${}^{c}$ 0.2099
							a${}_{CH_{2}\alpha }^{H}$(II)	0.9167
TiPrPD	a${}^{N}$	0.7169	DMeMorphA${}^{a}$	a${}^{N}$(I)	0.7088		a${}_{CH_{2}\beta }^{H}$(II)	0.6232
g-factor = 2.0059	a${}_{arom}^{H}$	(-) ${}^{c}$ 0.1864	g-factor = 2.0060	a${}_{arom}^{H}$(I)	(-) ${}^{c}$ 0.1979			
	a${}_{CH}^{H(1)}$	0.0961		a${}_{CH_{3}}^{H}$(I)	0.6935	BPipB	a${}^{N}$	0.7336
	a${}_{CH}^{H(2)}$	0.0784		a${}^{N}$(II)	0.7000	g-factor = 2.0059	a${}_{arom}^{H}$	(-) ${}^{c}$ 0.2342
	a${}_{CH_{3}}^{H}$	0.0179		a${}_{arom}^{H}$(II)	(-) ${}^{c}$ 0.2112		a${}_{CH_{2}\alpha }^{H}$	0.4382
				a${}_{CH_{2}\alpha }^{H}$(II)	0.5313		a${}_{CH_{2}\beta }^{H}$	0.0270
DMeMePiprzA ${}^{a}$	a${}^{N}$(I)	0.7111		a${}_{CH_{2}\beta }^{H}$(II)	0.0225		a${}_{CH_{2}\gamma }^{H}$	0.0211
g-factor = 2.0059	a${}_{arom}^{H}$(I)	(-) ${}^{c}$ 0.1936						
	a${}_{CH_{3}}^{H}$(I)	0.6809	BPyrB	a${}^{N}$	0.6849			
	a${}^{N}$(II)	0.7109	g-factor = 2.0059	a${}_{arom}^{H}$	(-) ${}^{c}$ 0.2032			
	a${}_{arom}^{H}$(II)	(-) ${}^{c}$ 0.2066		a${}_{CH_{2}\alpha }^{H}$	0.9675			
	a${}_{CH_{2}\alpha }^{H}$(II)	0.5250		a${}_{CH_{2}\beta }^{H}$	0.0206			
	a${}_{CH_{2}\beta }^{H}$(II)	0.0226						
	a${}_{\gamma }^{N}$(III)	0.1968						
	a${}_{CH_{3}\delta }^{H}$(III)	0.0200						

^a^ Concerning asymmetric compounds only, the central ring is divided as such; the methyl substituent branch is denoted (I), and the remaining branch is denoted (II). Unique to DMeMePiprzA, (III) represents the nitrogen and methyl group of the piperazin substituent. ^b^ The g-factor of DMeAzirA is not determined. ^c^ Negative values deduced from ENDOR analysis of $a_{arom}^{H}$ couplings in similar compounds.

**Table 3:** Calculated *R*${}^{2}$, slope, and intercept values with reference to B3LYP/6-31G(d,p) values. All results were generated using the PCM solvation model.

		$R^{2}$	Slope (*m*)	*Y*-Intercept (*c*)
B3LYP	6-31G(d,p)	0.8849	0.914	0.029
	6-31G(d,p)-J	0.8829	0.885	0.042
	6-311++G(d,p)-J	0.8787	0.889	0.045
	pcJ-1	0.8748	0.893	0.044
	pcJ-2	0.8662	0.894	0.048
	cc-pVTZ-J	0.8695	0.895	0.047

**Table 4:** Calculated *R*${}^{2}$, slope, and intercept values with reference to B3LYP/6-31G(d,p) values. All results were generated using the PCM solvation model.

		*R* ${}^{2}$	Slope (*m*)	*Y*-Intercept (*c*)
B3LYP	6-31G(d,p)	0.8849	0.914	0.029
TPSSh	6-31G(d,p)-J	0.8957	0.845	0.039
	6-311++G(d,p)-J	0.8773	0.857	0.047
	pcJ-1	0.8639	0.862	0.050
	pcJ-2	0.8621	0.864	0.053
	cc-pVTZ-J	0.8686	0.862	0.052
**PBE0**	**6-31G(d,p)-J**	**0.8926**	**0.873**	**0.039**
	6-311++G(d,p)-J	0.8913	0.877	0.040
	pcJ-1	0.8875	0.882	0.039
	pcJ-2	0.8862	0.884	0.044
	cc-pVTZ-J	0.8888	0.882	0.044

**Table 5:** DMeAzetA hfccs (mT) comparison between CFOUR without solvent, Gaussian without solvent, and Gaussian with solvent. Experimental results are provided as reference.

Coupling	CFOUR/No Solvent	Gaussian/No Solvent	Gaussian/Solvent	Experimental
a${}^{N}$(I)	1.2760	1.2749	0.7917	0.7200
a${}_{arom}^{H}$(I)	−1.7070	−1.7057	−0.6970	−0.2087
a${}_{CH_{3}}^{H}$(I)	1.0832	1.0822	0.8283	0.6541
a${}^{N}$(II)	−0.0780	−0.0799	0.3745	0.6791
a${}_{arom}^{H}$(II)	1.0973	1.0961	0.0815	−0.2099
a${}_{CH_{2}\alpha }^{H}$(II)	0.7367	0.7361	1.0702	0.9167
a${}_{CH_{2}\beta }^{H}$(II)	0.0071	0.0067	−0.0279	0.6232

**Table 6:** Linear regression analysis of DMeAzirA; three-member ring, DMeAzetA; four-member ring, PByrB; five-member ring and BPipB; six-member ring.

	Ring Number	$R^{2}$	Slope (*m*)	*Y*-Intercept (*c*)
DMeAzirA	3	0.3573	0.250	0.259
DMeAzetA	4	0.7399	0.661	0.160
BPyrB	5	0.9866	0.900	0.019
BPipB	6	0.9943	0.904	0.029

**Table 7:** DMeAzetA and DMeAzirA couplings absent; *R*${}^{2}$, slope, and intercept values are re-calculated without outliers and shown with reference to B3LYP/6-31G(d,p) values. All results were generated using the PCM solvation model.

		$R^{2}$	Slope (*m*)	*Y*-Intercept (*c*)
B3LYP	6-31G(d,p)	0.9514	0.998	0.003
TPSSh	6-31G(d,p)-J	0.9564	0.938	0.013
	6-311++G(d,p)-J	0.9356	0.952	0.022
	pcJ-1	0.9153	0.959	0.027
	pcJ-2	0.9166	0.962	0.030
	cc-pVTZ-J	0.9245	0.960	0.029
B3LYP	6-31G(d,p)-J	0.9549	0.991	0.016
	6-311++G(d,p)-J	0.9500	0.992	0.020
	pcJ-1	0.9408	0.997	0.021
	pcJ-2	0.9326	0.999	0.023
	cc-pVTZ-J	0.9370	1.001	0.023
**PBE0**	**6-31G(d,p)-J**	**0.9646**	**0.980**	**0.012**
	6-311++G(d,p)-J	0.9622	0.972	0.013
	pcJ-1	0.9534	0.978	0.014
	pcJ-2	0.9553	0.981	0.019
	cc-pVTZ-J	0.9582	0.980	0.019

**Table 8:** Linear regression analysis of MP2, PBE0, and ωB97XD for both problematic systems (DMeAzeta and DMeAzirA) with reference to a satisfactory model system (DMeDiPrPD).

		$R^{2}$	Slope (*m*)	*Y*-Intercept (*c*)
MP2	DMeAzetA	0.7944	0.603	0.245
Optimised MP2/6-31G(d,p)	DMeAzirA	0.8244	0.461	0.219
	DMeDiPrPD	0.9345	0.903	0.076
PBE0	DMeAzetA	0.7971	0.654	0.180
Optimised MP2/6-31G(d,p)	DMeAzirA	0.7990	0.719	0.079
	DMeDiPrPD	0.9493	0.944	0.005
ωB97XD	DMeAzetA	0.7782	0.689	0.175
Optimised MP2/6-31G(d,p)	DMeAzirA	0.9489	0.920	-0.046
	DMeDiPrPD	0.9556	0.998	-0.003
ωB97XD	DMeAzetA	0.7614	0.716	0.183
Optimised ωB97XD/6-31G(d,p)	DMeAzirA	0.7491	0.727	0.107
	DMeDiPrPD	0.9461	1.036	0.004

## Data Availability

The data that support the findings of this study are available within the article and the Supporting Information. Further data are available from the corresponding author upon reasonable request.

## Supplementary Materials

The supporting information can be downloaded at: https://www.mdpi.com/article/10.3390/ijms24043447/s1.

## Abbreviations

The following abbreviations are used in this manuscript:

hfcc	Hyperfine Coupling Constant
DFT	Density Functional Theory
B3LYP	Becke, Lee–Yang–Parr
TPSSh	Tao, Perdew, Staroverov, Scuseria, hybrid
PBE0	Perdew, Burke and Ernzerhof
