# Supplementary material for: A Combined Experimental and Theoretical Study of ESR Hyperfine Coupling Constants for N,N,N’,N’-Tetrasubstituted p-Phenylenediamine Radical Cations
Source: Int J Mol Sci. 2023 Feb 8;24(4):3447. doi: 10.3390/ijms24043447 (PMC9967363; doi:10.3390/ijms24043447)
Supplement: Supplementary file 1 [file ijms-24-03447-s001.zip › ijms-2173552-supplementary.pdf]

Supplementary Material
A Combined Experimental and Theoretical Study of ESR Hyperfine Coupling Constants for N,N,N',N'-Tetrasubstituted p-Phenylenediamine Radical Cations

Ronan Gleeson,[†] Cecilie L. Andersen,[†] Peter Rapta,[‡] Peter Machata,[‡,¶] Jørn B. Christensen,[†] Ole Hammerich,[†] and Stephan P. A. Sauer[*,†]

†*Department of Chemistry, University of Copenhagen, Universitetsparken 5, 2100 Copenhagen, Denmark*

‡*Institute of Physical Chemistry and Chemical Physics, Faculty of Chemical and Food Technology, Slovak University of Technology, Radlinského 9, 812 37 Bratislava, Slovak Republic*

¶*Present address at time of writing: Polymer Institute, Slovak Academy of Sciences, Dubravska cesta 9, Bratislava 845 41, Slovak Republic*

E-mail: sauer@chem.ku.dk

Table S1: Calculated hyperfine coupling constants (mT) for BMorphB

		B3LYP	TPSSh	PBE0
6-31G(d,p)	a^N	0.6438	-	-
	a^H_{arom}	-0.1722	-	-
	$a^H_{CH_2\alpha}$	0.5257	-	-
	$a^H_{CH_2\beta}$	0.0218	-	-
6-31G(d,p)-J	a^N	0.6056	0.6370	0.6413
	a^H_{arom}	-0.1755	-0.1869	-0.1801
	$a^H_{CH_2\alpha}$	0.5730	0.6070	0.5724
	$a^H_{CH_2\beta}$	0.0232	0.0367	0.0182
6-311++G(d,p)-J	a^N	0.5959	0.5906	0.6428
	a^H_{arom}	-0.1768	-0.1898	-0.1812
	$a^H_{CH_2\alpha}$	0.5948	0.6313	0.5913
	$a^H_{CH_2\beta}$	0.0146	0.0242	0.0096
pcJ-1	a^N	0.5822	0.5581	0.6246
	a^H_{arom}	-0.1745	-0.1874	-0.1791
	$a^H_{CH_2\alpha}$	0.6114	0.6474	0.6109
	$a^H_{CH_2\beta}$	0.0131	0.0236	0.0082
pcJ-2	a^N	0.5680	0.5523	0.6151
	a^H_{arom}	-0.1745	-0.1842	-0.1786
	$a^H_{CH_2\alpha}$	0.6065	0.6427	0.6037
	$a^H_{CH_2\beta}$	0.0141	0.0249	0.0094
cc-pVTZ-J	a^N	0.5729	0.5632	0.6204
	a^H_{arom}	-0.1749	-0.1852	-0.1796
	$a^H_{CH_2\alpha}$	0.6049	0.6427	0.6041
	$a^H_{CH_2\beta}$	0.0152	0.0257	0.0100

Table S2: Calculated hyperfine coupling constants (mT) for BPipB

		B3LYP	TPSSh	PBE0
6-31G(d,p)	a^N	0.6667	-	-
	a^H_{arom}	-0.1731	-	-
	$a^H_{CH_2\alpha}$	0.4509	-	-
	$a^H_{CH_2\beta}$	0.0166	-	-
	$a^H_{CH_2\gamma}$	0.0638	-	-
6-31G(d,p)-J	a^N	0.6366	0.6694	0.6736
	a^H_{arom}	-0.1784	-0.1907	-0.1830
	$a^H_{CH_2\alpha}$	0.4589	0.4821	0.4569
	$a^H_{CH_2\beta}$	0.0212	0.0292	0.0160
	$a^H_{CH_2\gamma}$	0.0734	0.0861	0.0730
6-311++G(d,p)-J	a^N	0.6166	0.6131	0.6660
	a^H_{arom}	-0.1798	-0.1932	-0.1843
	$a^H_{CH_2\alpha}$	0.4682	0.4926	0.4647
	$a^H_{CH_2\beta}$	0.0187	0.0252	0.0132
	$a^H_{CH_2\gamma}$	0.0695	0.0813	0.0683
pcJ-1	a^N	0.5971	0.5739	0.6412
	a^H_{arom}	-0.1736	-0.1864	-0.1780
	$a^H_{CH_2\alpha}$	0.4845	0.5090	0.4829
	$a^H_{CH_2\beta}$	0.0174	0.0247	0.0121
	$a^H_{CH_2\gamma}$	0.0701	0.0816	0.0695
pcJ-2	a^N	0.5780	0.5630	0.6268
	a^H_{arom}	-0.1711	-0.1801	-0.1751
	$a^H_{CH_2\alpha}$	0.5128	0.5403	0.5096
	$a^H_{CH_2\beta}$	0.0164	0.0228	0.0109
	$a^H_{CH_2\gamma}$	0.0652	0.0771	0.0647
cc-pVTZ-J	a^N	0.5914	0.5832	0.6409
	a^H_{arom}	-0.1778	-0.1888	-0.1824
	$a^H_{CH_2\alpha}$	0.4709	0.4960	0.4695
	$a^H_{CH_2\beta}$	0.0201	0.0276	0.0145
	$a^H_{CH_2\gamma}$	0.0704	0.0824	0.0697

Table S3: Calculated hyperfine coupling constants (mT) for BPyrB

		B3LYP	TPSSh	PBE0
6-31G(d,p)	a^N	0.6467	-	-
	a^H_{arom}	-0.1888	-	-
	$a^H_{CH_2\alpha}$	1.0329	-	-
	$a^H_{CH_2\beta}$	-0.0143	-	-
6-31G(d,p)-J	a^N	0.6042	0.6372	0.6384
	a^H_{arom}	-0.1862	-0.1964	-0.1918
	$a^H_{CH_2\alpha}$	1.1226	1.1989	1.1199
	$a^H_{CH_2\beta}$	-0.0156	-0.0188	-0.0194
6-311++G(d,p)-J	a^N	0.5928	0.5889	0.6391
	a^H_{arom}	-0.1860	-0.1974	-0.1916
	$a^H_{CH_2\alpha}$	1.1156	1.1860	1.1053
	$a^H_{CH_2\beta}$	-0.0160	-0.0194	-0.0198
pcJ-1	a^N	0.5771	0.5537	0.6188
	a^H_{arom}	-0.1817	-0.1935	-0.1871
	$a^H_{CH_2\alpha}$	1.1263	1.1976	1.1207
	$a^H_{CH_2\beta}$	-0.0167	-0.0198	-0.0204
pcJ-2	a^N	0.5605	0.5450	0.6069
	a^H_{arom}	-0.1840	-0.1918	-0.1891
	$a^H_{CH_2\alpha}$	1.1285	1.1970	1.1189
	$a^H_{CH_2\beta}$	-0.0157	-0.0191	-0.0198
cc-pVTZ-J	a^N	0.5645	0.5556	0.6106
	a^H_{arom}	-0.1844	-0.1932	-0.1905
	$a^H_{CH_2\alpha}$	1.1241	1.1963	1.1176
	$a^H_{CH_2\beta}$	-0.0158	-0.0192	-0.0198

Table S4: Calculated hyperfine coupling constants (mT) for DMeAzetA

		B3LYP	TPSSh	PBE0	MP2[a]	PBE0[b]	ωB97XD[c]
6-31G(d,p)	a^N(I)	0.6276	-	-	-	-	-
	a^H_{arom}(I)	-0.1633	-	-	-	-	-
	$a^H_{CH_3}$(I)	0.7011	-	-	-	-	-
	a^N(II)	0.6635	-	-	-	-	-
	a^H_{arom}(II)	-0.2171	-	-	-	-	-
	$a^H_{CH_2\alpha}$(II)	1.2341	-	-	-	-	-
	$a^H_{CH_2\beta}$(II)	-0.0459	-	-	-	-	-
6-31G(d,p)-J	a^N(I)	0.5814	0.6175	0.6137	0.7917	0.6442	0.6184
	a^H_{arom}(I)	-0.1614	-0.1754	-0.1626	-0.6970	-0.1897	-0.1713
	$a^H_{CH_3}$(I)	0.7641	0.8112	0.7615	0.8283	0.7914	0.7101
	a^N(II)	0.6244	0.6559	0.6611	0.3745	0.6731	0.6466
	a^H_{arom}(II)	-0.2144	-0.2218	-0.2243	0.0815	-0.1978	-0.1875
	$a^H_{CH_2\alpha}$(II)	1.3376	1.4242	1.3377	1.0702	1.4192	1.2706
	$a^H_{CH_2\beta}$(II)	-0.0512	-0.0573	-0.0575	-0.0279	-0.0620	-0.0562
6-311++G(d,p)-J	a^N(I)	0.5707	0.5700	0.6149	-	-	-
	a^H_{arom}(I)	-0.1626	-0.1773	-0.1638	-	-	-
	$a^H_{CH_3}$(I)	0.7617	0.8049	0.7539	-	-	-
	a^N(II)	0.6144	0.6088	0.6635	-	-	-
	a^H_{arom}(II)	-0.2126	-0.2217	-0.2225	-	-	-
	$a^H_{CH_2\alpha}$(II)	1.3287	1.4077	1.3199	-	-	-
	$a^H_{CH_2\beta}$(II)	-0.0510	-0.0572	-0.0575	-	-	-
pcJ-1	a^N(I)	0.5565	0.5379	0.5962	-	-	-
	a^H_{arom}(I)	-0.1579	-0.1731	-0.1587	-	-	-
	$a^H_{CH_3}$(I)	0.7687	0.8129	0.7646	-	-	-
	a^N(II)	0.5970	0.5720	0.6416	-	-	-
	a^H_{arom}(II)	-0.2085	-0.2178	-0.2184	-	-	-
	$a^H_{CH_2\alpha}$(II)	1.3418	1.4225	1.3378	-	-	-
	$a^H_{CH_2\beta}$(II)	-0.0529	-0.0585	-0.0593	-	-	-
pcJ-2	a^N(I)	0.5403	0.5291	0.5846	-	-	-
	a^H_{arom}(I)	-0.1617	-0.1726	-0.1623	-	-	-
	$a^H_{CH_3}$(I)	0.7716	0.8129	0.7640	-	-	-
	a^N(II)	0.5807	0.5634	0.6292	-	-	-
	a^H_{arom}(II)	-0.2095	-0.2151	-0.2187	-	-	-
	$a^H_{CH_2\alpha}$(II)	1.3453	1.4204	1.3360	-	-	-
	$a^H_{CH_2\beta}$(II)	-0.0521	-0.0583	-0.0587	-	-	-
cc-pVTZ-J	a^N(I)	0.5451	0.5401	0.5896	-	-	-
	a^H_{arom}(I)	-0.1624	-0.1748	-0.1647	-	-	-
	$a^H_{CH_3}$(I)	0.7702	0.8147	0.7651	-	-	-
	a^N(II)	0.5842	0.5740	0.6325	-	-	-
	a^H_{arom}(II)	-0.2094	-0.2158	-0.2192	-	-	-
	$a^H_{CH_2\alpha}$(II)	1.3389	1.4203	1.3350	-	-	-
	$a^H_{CH_2\beta}$(II)	-0.0519	-0.0588	-0.0589	-	-	-

[a] MP2 calculated hyperfine coupling values used in Figure 6 and Figure 7

[b] PBE0 calculated hyperfine coupling values used in Figure 7

[c] Reference calculation found in Table 8

Table S5: Calculated hyperfine coupling constants (mT) for DMeAzirA

		B3LYP	TPSSh	PBE0	MP2[a]	PBE0[b]	ωB97XD[c]
6-31G(d,p)	a^N(I)	0.7316	-	-	-	-	-
	a^H_{arom}(I)	-0.2804	-	-	-	-	-
	$a^H_{CH_3}$(I)	0.8289	-	-	-	-	-
	a^N(II)	1.1454	-	-	-	-	-
	a^H_{arom}(II)	-0.0767	-	-	-	-	-
	$a^{H(1)}_{CH_2}$(II)	0.5379	-	-	-	-	-
	$a^{H(2)}_{CH_2}$(II)	0.2751	-	-	-	-	-
6-31G(d,p)-J	a^N(I)	0.6799	0.7176	0.7299	0.2703	0.7265	0.6943
	a^H_{arom}(I)	-0.2769	-0.2850	-0.3109	0.3050	-0.2039	-0.1872
	$a^H_{CH_3}$(I)	0.9030	0.9538	0.9148	0.5450	0.8842	0.7884
	a^N(II)	1.2288	1.2097	1.2197	1.2900	0.9874	0.9672
	a^H_{arom}(II)	-0.0767	-0.0951	-0.0521	-0.902	-0.1774	-0.1658
	$a^{H(1)}_{CH_2}$(II)	0.5877	0.6164	0.5571	0.9080	0.9202	0.8519
	$a^{H(2)}_{CH_2}$(II)	0.3131	0.3519	0.3021	1.190	0.6894	0.6359
6-311++G(d,p)-J	a^N(I)	0.6689	0.6678	0.7321	-	-	-
	a^H_{arom}(I)	-0.2789	-0.2888	-0.3125	-	-	-
	$a^H_{CH_3}$(I)	0.9057	0.9518	0.9097	-	-	-
	a^N(II)	1.1936	1.1473	1.1940	-	-	-
	a^H_{arom}(II)	-0.0732	-0.0922	-0.0485	-	-	-
	$a^{H(1)}_{CH_2}$(II)	0.5748	0.5999	0.5429	-	-	-
	$a^{H(2)}_{CH_2}$(II)	0.2950	0.3307	0.2837	-	-	-
pcJ-1	a^N(I)	0.6455	0.6213	0.7029	-	-	-
	a^H_{arom}(I)	-0.2649	-0.2750	-0.2961	-	-	-
	$a^H_{CH_3}$(I)	0.9045	0.9517	0.9131	-	-	-
	a^N(II)	1.1679	1.1055	1.1694	-	-	-
	a^H_{arom}(II)	-0.0800	-0.0988	-0.0574	-	-	-
	$a^{H(1)}_{CH_2}$(II)	0.6403	0.6694	0.6103	-	-	-
	$a^{H(2)}_{CH_2}$(II)	0.3465	0.3847	0.3358	-	-	-
pcJ-2	a^N(I)	0.6296	0.6124	0.6911	-	-	-
	a^H_{arom}(I)	-0.2727	-0.2768	-0.3032	-	-	-
	$a^H_{CH_3}$(I)	0.9114	0.9531	0.9138	-	-	-
	a^N(II)	1.1477	1.0994	1.1553	-	-	-
	a^H_{arom}(II)	-0.0760	-0.0943	-0.0536	-	-	-
	$a^{H(1)}_{CH_2}$(II)	0.6073	0.6347	0.5783	-	-	-
	$a^{H(2)}_{CH_2}$(II)	0.3208	0.3623	0.3124	-	-	-
cc-pVTZ-J	a^N(I)	0.6325	0.6253	0.6945	-	-	-
	a^H_{arom}(I)	-0.2708	-0.2783	-0.3040	-	-	-
	$a^H_{CH_3}$(I)	0.9054	0.9525	0.9122	-	-	-
	a^N(II)	1.1712	1.1237	1.1750	-	-	-
	a^H_{arom}(II)	-0.0789	-0.0953	-0.0556	-	-	-
	$a^{H(1)}_{CH_2}$(II)	0.6142	0.6409	0.5838	-	-	-
	$a^{H(2)}_{CH_2}$(II)	0.3287	0.3689	0.3187	-	-	-

[a] MP2 calculated hyperfine coupling values used in Figure 6 and Figure 7

[b] PBE0 calculated hyperfine coupling values used in Figure 7

[c] Reference calculation found in Table 8

Table S6: Calculated hyperfine coupling constants (mT) for DMeDiPrPD

		B3LYP	TPSSh	PBE0	MP2[a]	ωB97XD[b]
6-31G(d,p)	a^{N}	0.6692	-	-	-	-
	$a^{H(1)}_{arom}$	-0.1918	-	-	-	-
	$a^{H(2)}_{arom}$	-0.1721	-	-	-	-
	$a^{H}_{CH_3}$(Me)	0.6906	-	-	-	-
	$a^{H}_{CH_3}$(iPr)	0.1609	-	-	-	-
	a^{H}_{CH}	0.5912	-	-	-	-
6-31G(d,p)-J	a^{N}	0.6226	0.6571	0.6580	0.5693	0.6352
	$a^{H(1)}_{arom}$	-0.1800	-0.1902	-0.1854	-0.2963	-0.1752
	$a^{H(2)}_{arom}$	-0.1800	-0.1902	-0.1854	-0.2974	-0.1752
	$a^{H}_{CH_3}$(Me)	0.7626	0.8053	0.7608	0.7083	0.7260
	$a^{H}_{CH_3}$(iPr)	0.0457	0.1612	0.1502	0.1325	0.1383
	a^{H}_{CH}	0.0470	0.0424	0.0528	0.0208	0.0834
6-311++G(d,p)-J	a^{N}	0.6103	0.6076	0.6579	-	-
	$a^{H(1)}_{arom}$	-0.1804	-0.1919	-0.1856	-	-
	$a^{H(2)}_{arom}$	-0.1804	-0.1919	-0.1856	-	-
	$a^{H}_{CH_3}$(Me)	0.7482	0.7872	0.7425	-	-
	$a^{H}_{CH_3}$(iPr)	0.0255	0.2062	0.1932	-	-
	a^{H}_{CH}	0.0323	0.0258	0.0380	-	-
pcJ-1	a^{N}	0.5925	0.5701	0.6352	-	-
	$a^{H(1)}_{arom}$	-0.1766	-0.1885	-0.1818	-	-
	$a^{H(2)}_{arom}$	-0.1766	-0.1885	-0.1818	-	-
	$a^{H}_{CH_3}$(Me)	0.7592	0.7992	0.7570	-	-
	$a^{H}_{CH_3}$(iPr)	0.0258	0.2077	0.1961	-	-
	a^{H}_{CH}	0.0321	0.0247	0.0378	-	-
pcJ-2	a^{N}	0.5769	0.5645	0.6265	-	-
	$a^{H(1)}_{arom}$	-0.1773	-0.1862	-0.1827	-	-
	$a^{H(2)}_{arom}$	-0.1773	-0.1862	-0.1827	-	-
	$a^{H}_{CH_3}$(Me)	0.7609	0.7997	0.7565	-	-
	$a^{H}_{CH_3}$(iPr)	0.0217	0.0222	0.0182	-	-
	a^{H}_{CH}	0.0382	0.0331	0.0442	-	-
cc-pVTZ-J	a^{N}	0.5829	0.5754	0.6305	-	-
	$a^{H(1)}_{arom}$	-0.1783	-0.1874	-0.1839	-	-
	$a^{H(2)}_{arom}$	-0.1783	-0.1874	-0.1839	-	-
	$a^{H}_{CH_3}$(Me)	0.7614	0.8014	0.7575	-	-
	$a^{H}_{CH_3}$(iPr)	0.0218	0.0222	0.0183	-	-
	a^{H}_{CH}	0.0413	0.0369	0.0474	-	-

[a] MP2 calculated hyperfine coupling values used in Figure 6

[b] Reference calculation found in Table 8

Table S7: Calculated hyperfine coupling constants (mT) for DMeDEtPD

		B3LYP	TPSSh	PBE0
6-31G(d,p)	a^N(I)	0.6374	-	-
	a^H_{arom}(I)	-0.1742	-	-
	$a^H_{CH_3}$(I)	0.7141	-	-
	a^N(II)	0.6693	-	-
	a^H_{arom}(II)	-0.2022	-	-
	$a^{H(1)}_{CH_2}$(II)	0.37	-	-
	$a^{H(2)}_{CH_3}$(II)	-0.0054	-	-
6-31G(d,p)-J	a^N(I)	0.5910	0.6243	0.6257
	a^H_{arom}(I)	-0.1722	-0.1830	-0.1770
	$a^H_{CH_3}$(I)	0.7785	0.8223	0.7783
	a^N(II)	0.6254	0.6618	0.6595
	a^H_{arom}(II)	-0.1992	-0.2106	-0.2060
	$a^{H(1)}_{CH_2}$(II)	0.4029	0.4235	0.4006
	$a^{H(2)}_{CH_3}$(II)	0.0613	0.0641	0.0558
6-311++G(d,p)-J	a^N(I)	0.5780	0.5746	0.6247
	a^H_{arom}(I)	-0.1716	-0.1834	-0.1761
	$a^H_{CH_3}$(I)	0.7733	0.8131	0.7676
	a^N(II)	0.6122	0.6120	0.6590
	a^H_{arom}(II)	-0.1993	-0.2123	-0.2059
	$a^{H(1)}_{CH_2}$(II)	0.3992	0.4185	0.3956
	$a^{H(2)}_{CH_3}$(II)	0.0603	0.0633	0.0549
pcJ-1	a^N(I)	0.5671	0.5452	0.6096
	a^H_{arom}(I)	-0.1702	-0.1822	-0.1750
	$a^H_{CH_3}$(I)	0.7858	0.8265	0.7840
	a^N(II)	0.5903	0.5702	0.6318
	a^H_{arom}(II)	-0.1922	-0.2054	-0.1983
	$a^{H(1)}_{CH_2}$(II)	0.3999	0.4195	0.3981
	$a^{H(2)}_{CH_3}$(II)	0.0598	0.0616	0.0549
pcJ-2	a^N(I)	0.5460	0.5321	0.5928
	a^H_{arom}(I)	-0.1693	-0.1775	-0.1733
	$a^H_{CH_3}$(I)	0.7812	0.8190	0.7759
	a^N(II)	0.5816	0.5686	0.6280
	a^H_{arom}(II)	-0.1971	-0.2065	-0.2033
	$a^{H(1)}_{CH_2}$(II)	0.4066	0.4252	0.4027
	$a^{H(2)}_{CH_3}$(II)	0.0611	0.0630	0.0556
cc-pVTZ-J	a^N(I)	0.5517	0.5433	0.5980
	a^H_{arom}(I)	-0.1708	-0.1796	-0.1755
	$a^H_{CH_3}$(I)	0.7812	0.8211	0.7775
	a^N(II)	0.5841	0.5795	0.6313
	a^H_{arom}(II)	-0.1963	-0.2070	-0.2035
	$a^{H(1)}_{CH_2}$(II)	0.4033	0.4239	0.4013
	$a^{H(2)}_{CH_3}$(II)	0.0614	0.0643	0.0559

Table S8: Calculated hyperfine coupling constants (mT) for DMeMePiprzA

		B3LYP	TPSSh	PBE0
6-31G(d,p)	a^N(I)	0.6305	-	-
	a^H_{arom}(I)	-0.1695	-	-
	$a^H_{CH_3}$(I)	0.7072	-	-
	a^N(II)	0.6744	-	-
	a^H_{arom}(II)	-0.1946	-	-
	$a^H_{CH_2\alpha}$(II)	0.5718	-	-
	$a^H_{CH_2\beta}$(II)	-0.0021	-	-
	a^N_γ(III)	0.0115	-	-
	$a^H_{CH_3\delta}$(III)	-0.0017	-	-
6-31G(d,p)-J	a^N(I)	0.5839	0.6158	0.6184
	a^H_{arom}(I)	-0.1667	-0.1764	-0.1717
	$a^H_{CH_3}$(I)	0.7700	0.8121	0.7700
	a^N(II)	0.6306	0.6655	0.6659
	a^H_{arom}(II)	-0.1928	-0.2048	-0.1985
	$a^H_{CH_2\alpha}$(II)	0.6244	0.6651	0.6211
	$a^H_{CH_2\beta}$(II)	-0.0011	0.0061	-0.0054
	a^N_γ(III)	-0.0123	-0.0106	-0.0148
	$a^H_{CH_3\delta}$(III)	-0.0017	-0.0015	-0.0021
6-311++G(d,p)-J	a^N(I)	0.5760	0.5720	0.6226
	a^H_{arom}(I)	-0.1711	-0.1819	-0.1766
	$a^H_{CH_3}$(I)	0.7716	0.8105	0.7660
	a^N(II)	0.6186	0.6155	0.6657
	a^H_{arom}(II)	-0.1883	-0.2019	-0.1932
	$a^H_{CH_2\alpha}$(II)	0.6434	0.6857	0.6370
	$a^H_{CH_2\beta}$(II)	-0.0050	-0.0009	-0.0096
	a^N_γ(III)	-0.0130	-0.0136	-0.0155
	$a^H_{CH_3\delta}$(III)	-0.0018	-0.0022	-0.0022
pcJ-1	a^N(I)	0.5610	0.5388	0.6032
	a^H_{arom}(I)	-0.1656	-0.1770	-0.1704
	$a^H_{CH_3}$(I)	0.7783	0.8179	0.7769
	a^N(II)	0.5964	0.5736	0.6390
	a^H_{arom}(II)	-0.1872	-0.2004	-0.1925
	$a^H_{CH_2\alpha}$(II)	0.6263	0.6653	0.6227
	$a^H_{CH_2\beta}$(II)	-0.0036	0.0029	-0.0083
	a^N_γ(III)	-0.0129	-0.0122	-0.0155
	$a^H_{CH_3\delta}$(III)	-0.0016	-0.0013	-0.0021
pcJ-2	a^N(I)	0.5443	0.5290	0.5902
	a^H_{arom}(I)	-0.1687	-0.1752	-0.1727
	$a^H_{CH_3}$(I)	0.7798	0.8156	0.7737
	a^N(II)	0.5853	0.5713	0.6332
	a^H_{arom}(II)	-0.1864	-0.1976	-0.1919
	$a^H_{CH_2\alpha}$(II)	0.6405	0.6821	0.6355
	$a^H_{CH_2\beta}$(II)	-0.0031	0.0027	-0.0077
	a^N_γ(III)	-0.0132	-0.0133	-0.0156
	$a^H_{CH_3\delta}$(III)	-0.0018	-0.0020	-0.0022
cc-pVTZ-J	a^N(I)	0.5474	0.5385	0.5935
	a^H_{arom}(I)	-0.1678	-0.1759	-0.1729
	$a^H_{CH_3}$(I)	0.7760	0.8148	0.7725
	a^N(II)	0.5897	0.5826	0.6379
	a^H_{arom}(II)	-0.1885	-0.1999	-0.1944
	$a^H_{CH_2\alpha}$(II)	0.6316	0.6741	0.6283
	$a^H_{CH_2\beta}$(II)	-0.0021	0.0039	-0.0069
	a^N_γ(III)	-0.0133	-0.0132	-0.0158
	$a^H_{CH_3\delta}$(III)	-0.0018	-0.0019	-0.0022

Table S9: Calculated hyperfine coupling constants (mT) for DMeMorphA

		B3LYP	TPSSh	PBE0
6-31G(d,p)	a^N(I)	0.6347	-	-
	a^H_{arom}(I)	-0.1752	-	-
	$a^H_{CH_3}$(I)	0.7126	-	-
	a^N(II)	0.6614	-	-
	a^H_{arom}(II)	-0.1875	-	-
	$a^H_{CH_2\alpha}$(II)	0.5322	-	-
	$a^H_{CH_2\beta}$(II)	0.0229	-	-
6-31G(d,p)-J	a^N(I)	0.5882	0.6177	0.6236
	a^H_{arom}(I)	-0.1724	-0.1805	-0.1789
	$a^H_{CH_3}$(I)	0.7762	0.8151	0.7770
	a^N(II)	0.6187	0.6552	0.6534
	a^H_{arom}(II)	-0.1858	-0.1987	-0.1901
	$a^H_{CH_2\alpha}$(II)	0.5811	0.6192	0.5783
	$a^H_{CH_2\beta}$(II)	0.0260	0.0409	0.0207
6-311++G(d,p)-J	a^N(I)	0.5841	0.5780	0.6319
	a^H_{arom}(I)	-0.1798	-0.1894	-0.1868
	$a^H_{CH_3}$(I)	0.7832	0.8194	0.7781
	a^N(II)	0.6016	0.6000	0.6476
	a^H_{arom}(II)	-0.1819	-0.1967	-0.1854
	$a^H_{CH_2\alpha}$(II)	0.5948	0.6346	0.5895
	$a^H_{CH_2\beta}$(II)	0.0157	0.0260	0.0105
pcJ-1	a^N(I)	0.5673	0.5432	0.6107
	a^H_{arom}(I)	-0.1729	-0.1833	-0.1791
	$a^H_{CH_3}$(I)	0.7877	0.8251	0.7870
	a^N(II)	0.5853	0.5644	0.6269
	a^H_{arom}(II)	-0.1818	-0.1960	-0.1858
	$a^H_{CH_2\alpha}$(II)	0.5889	0.6262	0.5863
	$a^H_{CH_2\beta}$(II)	0.0190	0.0314	0.0136
pcJ-2	a^N(I)	0.5501	0.5328	0.5970
	a^H_{arom}(I)	-0.1756	-0.1810	-0.1806
	$a^H_{CH_3}$(I)	0.7889	0.8217	0.7831
	a^N(II)	0.5713	0.5595	0.6187
	a^H_{arom}(II)	-0.1820	-0.1943	-0.1866
	$a^H_{CH_2\alpha}$(II)	0.5853	0.6241	0.5816
	$a^H_{CH_2\beta}$(II)	0.0194	0.0322	0.0143
cc-pVTZ-J	a^N(I)	0.5526	0.5418	0.5996
	a^H_{arom}(I)	-0.1743	-0.1814	-0.1805
	$a^H_{CH_3}$(I)	0.7839	0.8201	0.7808
	a^N(II)	0.5781	0.5722	0.6257
	a^H_{arom}(II)	-0.1841	-0.1963	-0.1890
	$a^H_{CH_2\alpha}$(II)	0.5850	0.6247	0.5828
	$a^H_{CH_2\beta}$(II)	0.0209	0.0336	0.0154

Table S10: Calculated hyperfine coupling constants (mT) for TiPrPD

		B3LYP	TPSSh	PBE0
6-31G(d,p)	a^N	0.6910	-	-
	a^H_{arom}	-0.1729	-	-
	$a^{H(1)}_{CH}$	0.1121	-	-
	$a^{H(2)}_{CH}$	0.1121	-	-
	$a^H_{CH_3}$	0.0041	-	-
6-31G(d,p)-J	a^N	0.6482	0.6806	0.6831
	a^H_{arom}	-0.1652	-0.1742	-0.1698
	$a^{H(1)}_{CH}$	0.0405	0.0364	0.0459
	$a^{H(2)}_{CH}$	0.1344	0.1274	0.1389
	$a^H_{CH_3}$	-0.0020	-0.0008	-0.0067
6-311++G(d,p)-J	a^N	0.6311	0.6277	0.6780
	a^H_{arom}	-0.1678	-0.1787	-0.1720
	$a^{H(1)}_{CH}$	0.0340	0.0288	0.0394
	$a^{H(2)}_{CH}$	0.1149	0.1068	0.1191
	$a^H_{CH_3}$	-0.0015	-0.0008	-0.0064
pcJ-1	a^N	0.6103	0.5857	0.6519
	a^H_{arom}	-0.1647	-0.1754	-0.1687
	$a^{H(1)}_{CH}$	0.0339	0.0280	0.0394
	$a^{H(2)}_{CH}$	0.1072	0.0986	0.1122
	$a^H_{CH_3}$	-0.0006	0.0007	-0.0055
pcJ-2	a^N	0.5993	0.5833	0.6457
	a^H_{arom}	-0.1647	-0.1727	-0.1688
	$a^{H(1)}_{CH}$	0.0341	0.0294	0.0397
	$a^{H(2)}_{CH}$	0.1159	0.1074	0.1200
	$a^H_{CH_3}$	-0.0011	0.0001	-0.0061
cc-pVTZ-J	a^N	0.6033	0.5949	0.6500
	a^H_{arom}	-0.1655	-0.1740	-0.1699
	$a^{H(1)}_{CH}$	0.0344	0.0302	0.0402
	$a^{H(2)}_{CH}$	0.1125	0.1050	0.1175
	$a^H_{CH_3}$	-0.0010	-0.0002	-0.0062

Table S11: Calculated hyperfine coupling constants (mT) for TMePD

		B3LYP	TPSSh	PBE0
6-31G(d,p)	a^N	0.6514	-	-
	a^H_{arom}	-0.1889	-	-
	$a^H_{CH_3}$	0.7317	-	-
6-31G(d,p)-J	a^N	0.6037	0.6385	0.6379
	a^H_{arom}	-0.1862	-0.1969	-0.1924
	$a^H_{CH_3}$	0.7968	0.8428	0.7947
6-311++G(d,p)-J	a^N	0.5917	0.5893	0.6385
	a^H_{arom}	-0.1857	-0.1974	-0.1917
	$a^H_{CH_3}$	0.7940	0.8358	0.7865
pcJ-1	a^N	0.5769	0.5556	0.6191
	a^H_{arom}	-0.1812	-0.1933	-0.1870
	$a^H_{CH_3}$	0.8015	0.8444	0.7979
pcJ-2	a^N	0.5595	0.5459	0.6063
	a^H_{arom}	-0.1838	-0.1918	-0.1893
	$a^H_{CH_3}$	0.8034	0.8432	0.7960
cc-pVTZ-J	a^N	0.5639	0.5568	0.6105
	a^H_{arom}	-0.1842	-0.1934	-0.1908
	$a^H_{CH_3}$	0.8011	0.8439	0.7960
